# Bird Migration Routes and Risk for Pathogen Dispersion into Western Mediterranean Wetlands

**DOI:** 10.3201/eid1303.060301

**Published:** 2007-03

**Authors:** Elsa Jourdain, Michel Gauthier-Clerc, Dominique Bicout, Philippe Sabatier

**Affiliations:** * Station Biologique de la Tour du Valat, Arles, France; † Ecole Nationale Vétérinaire de Lyon – Institut National de Recherche Agronomique, Marcy l'Etoile, France

**Keywords:** birds, migration, disease transmission, avian influenza, West Nile virus, perspective, wetlands, Mediterranean

## Abstract

Wild birds share with humans the capacity for moving fast over large distances. During migratory movements, birds carry pathogens that can be transmitted between species at breeding, wintering, and stopover places where numerous birds of various species are concentrated. We consider the area of the Camargue (southern France) as an example to highlight how ad hoc information already available on birds’ movements, abundance, and diversity can help assess the introduction and transmission risk for birdborne diseases in the western Mediterranean wetlands. Avian influenza and West Nile viruses are used as examples because birds are central to the epidemiology of these viruses.

## Abstract 2

Migratory movements of wild birds likely spread zoonotic infectious agents, such as avian influenza and West Nile viruses.

Birds are the only terrestrial vertebrates that share with humans the peculiarity of traveling in a few hours across national and intercontinental borders. The record for distance covered in a single year belongs to the arctic tern (*Sterna paradisaea*), which travels ≈50,000 km between Antarctica and northern Scandinavia. As a whole, billions of birds travel between continents twice a year in only a few weeks (*1*). During these yearly migrations, birds have the potential of dispersing microorganisms that can be dangerous for public as well as animal health (*2*,*3*). For instance, birds are believed to be responsible for the wide geographic distribution of various pathogens, including viruses (e.g., West Nile, Sindbis, influenza A, Newcastle), bacteria (e.g., borrelia, mycobacteria, salmonellae), and protozoa (e.g., cryptosporidia). Insight into the ecology of bird populations is necessary to understand the epidemiology of bird-associated diseases. Furthermore, data about avian movements might be used to improve disease surveillance schemes or to adapt preventive measures. However, solid bridges between ecology and human medicine are still lacking.

We explored the bird sector, in an attempt to provide general ideas on bird abundance, migration, geographic origin, and interspecies mingling. We focused on the Camargue area, an alluvial lowland covering some 140,000 ha in the Rhône Delta. As other Mediterranean wetlands (Figure 1), the Camargue is a major rallying point for Palearctic birds that are migrating between the great continental masses of Eurasia and Africa. This area is the current focus of intense sampling to study 2 pathogens closely associated with wild birds: avian influenza (AI) virus and West Nile virus (WNV). These 2 viruses have very different transmission cycles and ecology: AI viruses have a waterborne transmission, and ducks are their main natural reservoirs (*4*–*8*); WNV has a vectorborne transmission, and passerines are believed to play a major role in the amplification cycle (*9*–*11*). However, both viruses are known to be carried by reservoir birds during migration and have been associated with emerging disease transmission risk for humans and domestic animals (*2*,*5*,*7*,*11*,*12*). For both of them, the avifauna abundance, diversity, and departure origin may be of key importance in terms of disease transmission. We use these 2 viruses as examples in our discussion of the risk for dispersion of bird-carried pathogens into Mediterranean wetlands.

**Figure 1:**
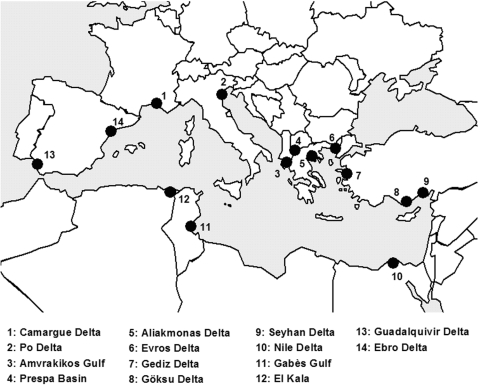
Map of the main Mediterranean wetlands (sites no. 1, 2, 11, 12, 13, 14 are considered western Mediterranean wetlands).

We address the following questions: 1) What are the main geographic origins of birds observed in western Mediterranean wetlands? 2) How abundant and diverse in species are they during the year cycle? 3) When are interspecies contacts between birds from different origins most likely to occur? To address these issues, we used crude empiric indexes, which are known to have biases yet prove valuable within the scope of our objectives. Readers interested in modern ecologic methods used to study wildlife diseases in natural populations may refer to general publications on host-parasite systems (*13*–*15*).

## Methods

### Bird Origins from Individual Ringing

Migration research is constantly changing, and new methods are always emerging. Historically, information about the movements of individual birds was first acquired through ringing studies. Bird ringing (also known as bird banding) consists of catching birds and attaching a small individually numbered metal or plastic ring to their legs or wings. Ring-recovery data are obtained when ringed birds are resighted, recaptured, hunted, or found dead. In Europe, large-scale ringing projects have been conducted, mostly between the 1950s and 1980s, and they represent a wealth of information that has not yet been fully exploited.

Data recovered from birds ringed from 1950 to 1975 at the Station Biologique de la Tour du Valat in the Camargue were collected from annual reports. Seven species of waterbirds were chosen to illustrate various migratory patterns. We selected 4 species of the Anatidae family, known to have different geographic origins, including 3 dabbling ducks, i.e., ducks that search for their food primarily in surface water (mallard, *Anas platyrhynchos*, n recovered = 434; green-winged teal, *A. crecca*, n = 3,903; garganey, *A. querquedula*, n = 181) and 1 diving duck, i.e., a species that mostly searches for its food under water (tufted duck, *Aythya fuligula*, n = 313). We also took the example of the common coot (*Fulica atra*, n = 99), a diving bird of the Rallidae family that frequently shares ponds with ducks. The common snipe (*Gallinago gallinago*, n = 54) is an example among waders, i.e., shorebirds that feed in muddy swamps and coastlines. Finally, the purple heron (*Ardea purpurea*, n = 39) is an ardeid species that lives in reed beds and marshes. All these species are large or hunted, which explains the high number of rings recovered. We only considered data recovered from birds ringed in the Camargue area and later reported outside France.

### Migratory Bird Abundance and Diversity

Since the 1950s, a large amount of data have been collected at the Station Biologique de la Tour du Valat thanks to bird counts, netting records, and field ornithologists’ observations (see supplemental, online technical Appendix l for table indicating the methods used for each bird genus; available from www.cdc.gov/EID/content/13/3/zzz_app1.htm). This information was used to create a database with a line for each of the 289 avian species regularly observed in the Camargue (*16*). Strictly pelagic birds were not taken into account as they do not have any contact with terrestrial vertebrate species. Quantitative data were completed on the number of birds (abundance) and number of bird species (diversity) observed monthly in the Camargue. Three categories of migrating birds were considered, depending on the area from which they come: incoming birds from sub-Saharan Africa in spring and those arriving in autumn either from continental Europe or from Scandinavian and the Siberian tundra and taiga. Analyses were performed for all species and separately for species of the Anatidae family (ducks, swans, geese) and waders (shorebirds of the families Scolopacidae and Charadriida*e*), which are essentially associated with wetlands or coastlines.

### Interspecies Bird Cohabitation

Regular bird counts provide information on bird populations for the studied area and therefore give an idea of potential contacts between species that share similar biotopes. Since September 1964, the Camargue duck and coot populations have been estimated every winter (*17*). The count was made monthly by the same observer from a plane flying at an altitude of 200 feet. One hundred brackish lakes and marshes used by waterbirds as resting places were counted. The arrival of the plane made dabbling ducks fly off, which is necessary for detecting them and identifying their species. To count diving ducks, it was necessary to turn the group of birds around by using the plane. Results of the winter 2004–05 counts were used as examples.

## Results

### Bird Origins from Individual Ringing

**Figure 2:**
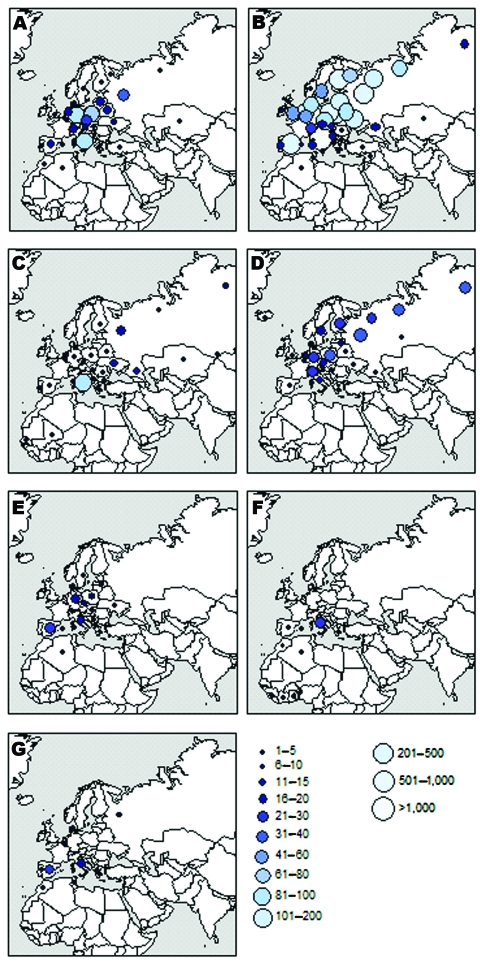
Countries and regions of the former USSR where birds ringed in the Camargue were recaptured for 7 species (n = number of ring recoveries and m = number of marked individual birds): A) mallard (*Anas platyrhynchos*), n = 434, m = 13,176; B) green-winged teal (*A. crecca*), n = 3,903, m = 58,347; C) garganey (*A. querquedula*), n = 181, m = 2,436; D) tufted duck (*Aythya fuligula*), n = 313, m = 3,845; E) common coot (*Fulica atra*), n = 99, m = 7,866; F) purple heron (*Ardea purpurea*), n = 39, m = 5,017; G) common snipe (*Gallinago gallinago*), n = 54, m = 2,445. These maps provide an insight into the potential origins and dispersion areas of birdborne pathogens.

Ringing recoveries provide a valuable insight into the origins and dispersion areas of bird species. Figure 2 illustrates that western Mediterranean wetlands provide habitat for birds from a wide geographic range: all European countries but also other areas in the Mediterranean Basin, central and northern Asia, and sub-Saharan Africa. Ringed common coots and common snipes were mostly reported from continental Europe and Mediterranean areas, whereas mallards and common teals were also found in more northern places, including the former Soviet Union and Scandinavia. The pattern was slightly different for tufted ducks, for which >40% of recoveries were located in areas of taiga and tundra. Garganeys were recaptured in very distant places far north (Siberia, Finland), far east (Kazakhstan, Altai), and far south (Senegal, Mali) of the Camargue. In contrast to the previously described species, purple heron rings were recovered only from areas located south, including 4 countries in the Guinea Gulf in Africa (Benin, Côte d’Ivoire, Ghana, and Sierra Leone). As a whole, we discerned 3 broad areas from which Mediterranean waterbirds come and potentially disperse pathogens: continental Europe, northern Siberia and Scandinavia, and sub-Saharan Africa.

### Migratory Bird Abundance and Diversity

**Figure 3:**
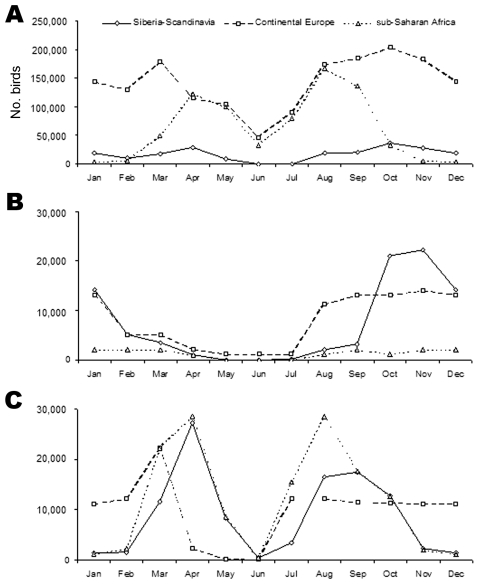
Monthly abundance in the Camargue of birds coming from Siberia/Scandinavia, continental Europe, and sub-Saharan Africa for A) all species, B) species of the Anatidae family and C) waders, respectively. Peaks in bird abundance are expected to be associated with a higher probability of dispersing birdborne pathogens into the Camargue.

**Figure 4:**
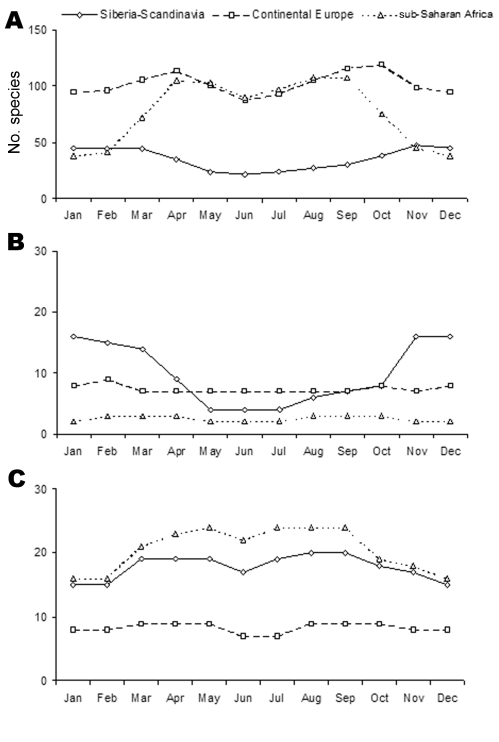
Monthly diversity in the Camargue of birds coming from Siberia/Scandinavia, continental Europe, and sub-Saharan Africa for A) all species, B) species of the Anatidae family, and C) waders, respectively. Peaks in bird species diversity are expected to be associated with a higher diversity of birdborne pathogens.

Monthly abundance (number of individual birds) and diversity (number of species) in the Camargue are presented respectively in Figures 3 and 4 for birds originating from the 3 major areas of provenance described above. These figures show how many birds are in the Camargue, just as monthly photographs of bird populations do. A corresponding table indicates monthly abundance of each species (Technical Appendix 1).

#### Birds Coming from sub-Saharan Africa

As many as 111 bird species might disperse pathogens from sub-Saharan Africa into the Camargue. Broadly speaking, birds coming from sub-Saharan Africa become rapidly and simultaneously abundant and diverse in spring, are still numerous in summer, and decrease in winter. The pattern is different if one considers solely ducks, as only 3 duck species fly south to tropical Africa, namely, the northern pintail (*Anas acuta*), the garganey, and the northern shoveler (*Anas clypeata*). Conversely, numbers and species diversity are high for waders, which are mainly passage visitors, especially in spring and late summer.

#### Birds Coming from Northern Areas of Tundra and Taiga

A total of 53 species might introduce pathogens from northern areas into the Camargue. Abundance is highest in April and October–November with a higher peak in autumn, notably because of juvenile birds. Species diversity is high during winter and low from May to July. The opposite pattern was observed for sub-Saharan species. This pattern is even clearer for birds of the Anatidae family: They are abundant from October to January and in very small numbers from March to September. In contrast to ducks, waders are mainly transient visitors, and only a few individual birds spend the winter in the Camargue. Their number is greatest in spring and autumn.

#### Birds Coming from Continental Europe

Up to 153 species might be involved in pathogen dispersion from continental Europe. Their abundance is highest from February to April and later from September to November. Species diversity remains high year-round with peaks in spring and autumn due to migrating passage visitors. The pattern observed for *Anatidae* species is the same as the 1 we described for Arctic species: birds are abundant in autumn and winter and in very small numbers in spring and early summer. However, the number of duck species remains stable year-round. Indeed, in species such as the mallard or the red-crested pochard (*Netta rufina*), some birds are sedentary whereas others are migratory. Waders show a constant level of species diversity because migration staggers over several months, but numbers are highly variable throughout the year.

### Interspecies Bird Cohabitation

**Figure 5:**
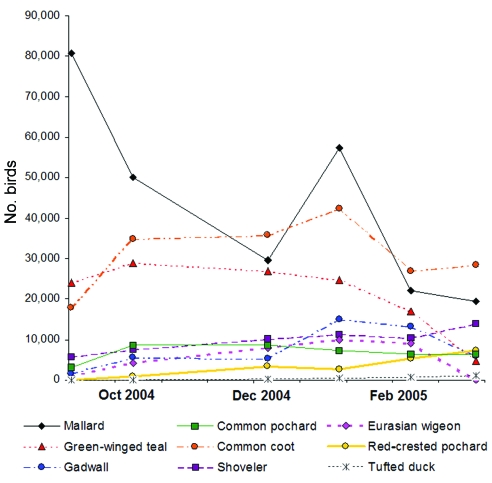
Cumulative number of the most abundant waterfowl species recorded in the Camargue during winter 2004–05: mallard (*Anas platyrhynchos*), northern shoveler (*A. clypeata*), green-winged teal (*A. crecca*), Eurasian wigeon (*A. penelope*), gadwall (*A. strepera*), red-crested pochard (*Netta rufina*), common pochard (*Aythya ferina*), tufted duck (*Aythya fuligula*), and common coot (*Fulica atra*). This figure shows that numerous birds and bird species are congregated in the same wetlands during winter and can therefore easily transmit pathogens to each other.

The results of the winter 2004–05 waterfowl counts are presented in Figure 5 for the species mentioned in Methods. Other species are also present, such as the northern pintail or the common shelduck (*Tadorna tadorna*). Garganeys are present in small numbers in September and February–March, but from an airplane they cannot be distinguished from common teals. These counts show that numerous species, with various migratory patterns, congregate on the same wetlands during the long winter period and therefore easily transmit waterborne pathogens such as AI virus. Most wintering birds are still present in March, when the first African migratory birds have already returned to breed in the Camargue or make a stop for refueling before flying further north. For instance, as many as 11,550 black-tailed godwits (*Limosa limosa*) were counted in the Camargue in March 2005.

Moreover, the movement and abundance of birds vary greatly from 1 year to another because of movements influenced by weather conditions. For example, the duck population in the Camargue was estimated at ≈60,000 ducks in March 2005 compared with only ≈40,000 the previous year, when climatic conditions in Europe were warmer.

## Discussion

Maps of ring-recovery data and graphs of monthly variations in bird abundance and diversity show that western Mediterranean wetlands such as the Camargue are a hub for birds from all origins (Central Asia, Siberia, northern and Eastern Europe, western Africa, and the Mediterranean basin) and that numerous birds of various species are seasonally aggregated in similar habitats. Under the hypothesis that risk for dispersion of pathogens into the Camargue is correlated with the number of birds and bird species encountered in a given area, these indices are helpful to determine periods at higher risk for introduction and emergence of birdborne diseases. We recall that these empiric estimates are skewed, which is briefly discussed with the perspectives below.

### Periods of Higher Risk for Pathogen Introduction

#### Birds Coming from sub-Saharan Africa

The risk for introduction of African pathogens in Mediterranean wetlands would be highest from March to July, which corresponds with spring migration and breeding for birds. Conversely, in autumn, birds return to Africa and are more likely to introduce pathogens originating from the north than from the south (Table). Of the 111 species that come every year to the Camargue from different countries of sub-Saharan Africa, most are insectivorous passerines that spend winter in Africa and breed in Europe; among aquatic birds, waders are the most numerous.

**Table:** Periods of major risk for pathogen introduction in the Camargue from sub-Saharan Africa, Siberia-Scandinavia, or continental Europe for all species, Anatidae species, and waders*

Origin	Species	Spring	Summer	Autumn	Winter
sub-Saharan Africa	All	++	+	(±)	(–)
	Waterfowl	+/–	–	(±)	(–)
	Waders	++	+	(+)	(–)
Scandinavia/Siberia	All	(±)	(–)	++	+
	Waterfowl	(–)	(–)	++	++
	Waders	(+)	(±)	++	±
Continental Europe	All	(±)	(–)	++	+
	Waterfowl	(–)	(–)	++	++
	Waders	(+)	(–)	++	+

*The risk is supposed to increase both with the number of species and the number of individual birds present in the Camargue (++, very high; +, high; ±, medium; –, low). In addition, the timing of migration matters since the introduction of pathogens from Africa (Eurasia) during the autumn (spring) migration is less likely because birds do not come directly from these areas. The corresponding risks are therefore in parentheses.

Up to now, no evidence exists that birds migrating from sub-Saharan regions play a major part in the epidemiology of AI viruses. However, under the assumption that this area became an important epicenter for AI viruses, ducks would likely have the highest probability of introducing AI viruses in Mediterranean wetlands, even if they are less numerous than waders. Indeed, recent studies in Europe showed that overall AI virus prevalence in waders is really low compared with that in dabbling ducks (*7*). WNV, which is transmitted by arthropod vectors, could potentially be introduced by any species of bird that comes from disease-endemic areas in Africa, is exposed to mosquito or tick bites (*18*), and sustains high viremia levels. Insectivorous passerines are the most numerous and thus may be particularly suspected. WNV dispersion by birds migrating from sub-Saharan Africa might explain why an outbreak occurred in 2000 in the Camargue, even though the virus had not been observed there since the 1960s (*19*).

#### Birds Coming from Northern Areas of Tundra and Taiga

Pathogens may be introduced into Mediterranean wetlands by birds coming from northern areas of Scandinavia and Siberia. The risk would be higher from September to December when Arctic bird abundance reaches its peak (Table). In spring, the northern birds observed in the Camargue have recently spent a long time in southern lands so that their associated probability of introducing pathogens originating from Scandinavia or Siberia is rather low. Waterbirds and granivorous passerines, which do not need to fly further south to find food supplies throughout the cold season, could introduce pathogenic microorganisms that could be transmitted later between wintering birds when densities are high. Waders, which migrate from Siberia and stop in the Mediterranean wetlands in autumn before crossing the Mediterranean Sea, could contaminate other bird species before pursuing their flight. As a whole, 53 species seen in the Camargue come from Arctic areas, which is half the number of species that come from sub-Saharan Africa or continental Europe. As a result, the probability of pathogens being introduced from Arctic areas should be lower than from birds of these 2 other areas. Another scenario can nevertheless be considered: if birds coming from northern areas disseminate a pathogen all along their migration route, then this pathogen would also infect continental European species and the probability of its being introduced into the Mediterranean wetlands would depend on the arrival of both Arctic and continental birds.

AI viruses are likely to be introduced in autumn by ducks that breed in northern Europe and Siberia, especially since numbers are high because of the presence of juveniles. Furthermore, surveillance studies of wild ducks showed that the prevalence of AI viruses is primarily high in juveniles (*5*,*7*,*20*). Conversely, WNV activity has never been reported in Scandinavia and Siberia, probably because the transmission cycle cannot be maintained in these northern biotopes.

#### Birds Coming from Continental Europe

Autumn and winter are the 2 seasons during which the transmission of bird pathogens originating from continental Europe would be most likely (Table). Indeed, in spring, the introduction of pathogens from continental Europe is less probable because birds have been absent from this area for 5 or 6 months. As previously seen, up to 135 species have the potential to introduce pathogenic agents in the Camargue. Granivorous passerines, birds of prey, and waterfowl are among the species that come in large numbers to take advantage of the Mediterranean wetlands’ temperate climate during winter. Aquatic birds, which need unfrozen ponds to feed, show variations in their movements, depending on climatic conditions. For instance, if a cold spell occurs in eastern or northern Europe, the number of green-winged teals in the Camargue increases (*17*). These weather-associated movements might at certain times prove essential in pathogen dispersion within European and Mediterranean wetlands.

Surveys of wild waterbirds in Europe have shown that AI viruses are frequently found (*21*–*24*), which means that waterbirds arriving from continental Europe might often be carriers of AI viruses. Similarly, since WNV activity was recently reported in Romania (*25*) and the Czech Republic (*26*), wild birds migrating in autumn from these countries to the Mediterranean basin could introduce WNV, either because of a high viremia level or because they carry infected ectoparasites. If the virus managed to overwinter in a reservoir host or a vector, it could then be responsible for an outbreak the next summer, when mosquito vectors are abundant (*27*).

### Risk for Bird-to-Bird Transmission of Pathogens

Several factors affect the risk for bird-to-bird transmission: bird abundance or density, bird diversity, species receptivity and sensitivity to pathogens, interspecies interactions, and environmental conditions (*14*). For water-transmitted pathogens such as AI viruses, risk for transmission may be associated with the number of ducks congregated in the same pond, particularly in autumn and winter (Figure 5). This crowding of wintering species, in addition to the permanent presence of a transient population of birds using wetlands to stop off during migration, could allow AI viruses to circulate and be maintained because of rapid dissemination on shared water. For vector-transmitted pathogens such as WNV, transmission possibilities depend both on the bird reservoir density and on the dispersion abilities and activity periods of the arthropod vectors.

The risk for interspecies transmission of disease is particularly problematic when wild and domestic species are involved. Ducks are aquatic birds that are most likely to come in contact with free-range poultry, especially because the presence of congeners can induce migrating wild ducks to make a stopover. Captive-bred mallards, used for hunting purposes and voluntarily put in the wild to attract other ducks, are particularly likely to share pathogens with their migratory congeners and facilitate the transmission of diseases to other domestic species. The risk is different for domestic chickens or turkeys, which are more likely to have contact with granivorous birds. Conversely, waders are rarely in direct contact with human-raised species.

Bird-carried pathogens are above all susceptible to being spread worldwide because of human activities such as legal or illegal trade of wild and domestic birds or bird products (*28*). The mechanism for the introduction of WNV into America in 1999 is not known with certainty, but a plausible scenario is the importation of an infected bird (*29*,*30*). Similarly, the highly pathogenic AI strain H5N1 was isolated in Belgium from crested hawk-eagles (*Spizaetus nipalensis*) smuggled by air travel (*31*). In Asia, transmission of H5N1 influenza virus has mainly been the result of human activity such as live-poultry markets and the international trade of birds, bird products, or contaminated equipment (*32*–*35*).

### Methodologic Concerns and Perspectives

The ornithologic data we have presented are merely crude estimates. Ring-recovery data, for instance, are subject to strong biases related to where and when the ringing was conducted but also to high variability in the probability of reporting marked animals among areas (*36*). Similarly, our estimates of bird abundance and diversity are basic indices associated with the number of birds heard, seen, or caught in the Camargue (Technical Appendix 2). These estimates do not take into account 2 important sources of error: detection error, related to the fact that the probability of detecting a bird is <1, and survey error, associated with spatial and temporal variability (*37*). Since our motivations were merely to show that information already available on birds may lead to better understanding of animal and human health issues associated with birdborne pathogens, these biases do not invalidate our objectives.

The results obtained were helpful to identify key groups of species likely to introduce pathogens from a given area at a given time of year. We voluntarily chose to focus on birds and leave pathogens aside, but studies of diseases in natural bird populations are obviously critically needed. Ecology, the science of interactions between living organisms and their physical environment, has been extended to include microorganisms. Understanding the relationships between organisms (such as hosts, pathogens, predators, competitors) and their environment is the aim of disease ecology. As studying the dynamics of systems with many hosts and pathogenic agents is complex, efforts should primarily focus on a few specific bird-pathogen models.

Mathematical modeling may help to predict specific bird-pathogen interactions and to identify key parameters that need to be better estimated through additional research. Long-term records enable establishment of databases, which would illustrate bird-pathogen relationships in natural conditions. These data would focus on hosts, their migration, population age, behavior, and so forth. Host-pathogen interactions should be described by using data such as antibody prevalence in different age classes, frequency of virus isolation, and characterization of the strains involved. Complementary laboratory and field experiments within a controlled environment might also provide relevant information. All these investigations should gradually make it possible to gather valuable baseline data to test specific hypotheses and gain new insights in bird-pathogen relationships in Mediterranean wetlands.

## Supplementary Material

Technical Appendix 1 Census methods used since 1970s for each bird genus in the Camargue*

Technical Appendix 2 P. Bird migration routes and risk for pathogen dispersion into western Mediterranean wetlands.
