# Supplementary material for: Bird Migration Routes and Risk for Pathogen Dispersion into Western Mediterranean Wetlands
Source: Emerg Infect Dis. 2007 Mar;13(3):365–72. doi: 10.3201/eid1303.060301 (PMC2725901; doi:10.3201/eid1303.060301)
Supplement: Technical Appendix 1 — Census methods used since 1970s for each bird genus in the Camargue* [file 06-0301_Techapp1-s1.pdf]

Jourdain E, Gauthier-Clerc M, Bicout D, Sabatier P. Bird migration routes and risk for pathogen dispersion into western Mediterranean wetlands. Emerg Infect Dis. 2007 Mar. Available from http://www.cdc.gov/EID/content/13/3/365.htm

Technical Appendix Table 1. Census methods used since 1970s for each bird genus in the Camargue*

Family	Genus	A	B	C	D	E	F	G	H	I	J	K	L	M	N
GAVIIDAE	*Gavia*	x	x	x	x							x			
PODICIPEDIDAE	*Tachybaptus, Podiceps*	x	x	x	x							x			
PROCELLARIIDAE	*Calonectris, Puffinus*														
HYDROBATIDAE	*Hydrobates*														
SULIDAE	*Morus*														
PHALACROCORACIDAE	*Phalacrocorax*	x	x	x	x	x									
ARDEIDAE	*Botaurus*	x	x	x								x			x
	Ixobrychus	x	x	x								x			x
	Ardeola, Nycticorax, Egretta, Ardea, Bubulcus	x	x	x	x	x		x	x						x
CICONIIDAE	*Ciconia*	x	x	x	x										
THRESKIORNITHIDAE	*Plegadis, Threskiornis*	x	x	x	x	x		x							
	Plataela	x	x	x	x		x	x	x						
PHŒNICOPTERIDAE	*Phoenicopterus*	x	x	x	x		x	x							
ANATIDAE	*Cygnus*	x	x	x	x		x		x						
	Anser, Aythya	x	x	x	x		x								
	Tadorna, Anas	x	x	x	x		x					x			
	Netta	x	x	x	x		x								x
	Somateria, Clangula, Melanitta, Bucephala, Mergus	x	x	x											
ACCIPITRIDAE	*Pernis, Milvus, Neophron*	x	x	x	x					x		x			
	Circaetus, Circus, Accipiter, Buteo	x	x	x	x					x		x			
	Aquila, Hieraaetus	x	x	x	x					x					
PANDIONIDAE	*Pandion*	x	x	x	x					x					
FALCONIDAE	*Falco*	x	x	x	x					x		x			
PHASIANIDAE	*Alectoris, Coturnix, Phasianus*	x	x	x	x							x			
RALLIDAE	*Rallus, Gallinula*	x	x	x	x							x			
	Crex, Porzana	x	x	x	x										
	Fulica	x	x	x	x		x					x			
GRUIDAE	*Grus*	x	x	x	x		x								
HAEMATOPODIDAE	*Haematopus*	x	x	x	x										
RECURVIROSTRIDAE	*Himantopus, Recurvirostra*	x	x	x	x										
BURHINIDAE	*Burhinus*	x										x			
GLAREOLIDAE	*Glareola*	x	x	x	x										x
CHARADRIIDAE	*Charadrius, Pluvialis, Vanellus, Xenus*	x	x	x	x										
SCOLOPACIDAE	*Limosa, Numenius*	x	x	x	x		x								

This material, provided by the author(s) as a supplement to Bird Migration Routes and Risk for Pathogen Dispersion into Western Mediterranean Wetlands, is not part of Emerging Infectious Diseases contents.

Family	Genus	A	B	C	D	E	F	G	H	I	J	K	L	M	N
	Scolopax	x											x		
	Gallinago, Lymnocryptes	x	x	x	x								x		
	Calidris, Limicola, Tringa, Actitis, Philomachus, Phalaropus, Phalaropus, Arenaria	x	x	x	x										
STERCORARIIDAE	*Catharacta, Stercorarius*	x	x	x	x										
LARIDAE	*Larus, Sterna, Gelochelidon, Chlidonias*	x	x	x	x	x		x							x
ALCIDAE	*Alca, Fratercula*														
PTEROCLIDIDAE	*Pterocles*	x													
COLUMBIDAE	*Streptopelia, Columba*	x										x			
CUCULIDAE	*Cuculus, Clamator*	x										x			
STRIGIDAE	*Tyto, Otus, Asio, Bubo, Strix, Athene*	x										x			
CAPRIMULGIDAE	*Caprimulgus*	x										x			
APODIDAE	*Apus*	x													
ALCEDINIDAE	*Alcedo*	x													
MEROPIDAE	*Merops*	x													x
CORACIIDAE	*Coracias*	x										x			
UPUPIDAE	*Upupa*	x										x			
PICIDAE	*Jynx*	x										x			
	Picus, dendrocops	x										x			x
ALAUDIDAE	*Galerida, Lullula, Alauda, Calandrella, Melanocorypha*	x								x	x	x	x	x	
HIRUNDINIDAE	*Riparia, Hirundo, Delichon*	x								x	x	x	x	x	x
MOTACILLIDAE	*Anthus, Motacilla*	x								x	x	x	x	x	
CINCLIDAE	*Cinclus*	x								x	x	x	x	x	
TROGLODYTIDAE	*Troglodytes*	x								x	x	x	x	x	
PRUNELLIDAE	*Prunella*	x								x	x	x	x	x	
TURDIDAE	*Oenanthe, Saxicola, Phoenicurus*	x								x	x	x	x	x	
	Erithacus, Turdus, Monticola	x								x	x	x	x	x	
	Luscinia	x								x	x	x	x	x	x
SYLVIIDAE	*Acrocephalus*	x								x	x	x	x	x	x
	Locustella, Cisticola	x								x	x	x	x	x	
	Regulus, Phylloscopus, Hippolais, Sylvia, Cettia	x								x	x	x	x	x	
MUSCICAPIDAE	*Ficedula, Muscicapa*	x								x	x	x	x	x	
TIMALIIDAE	*Panurus*	x								x	x	x	x	x	
AEGITHALIDAE	*Aegithalos*	x								x	x	x	x	x	
PARIDAE	*Parus*	x								x	x	x	x	x	
SITTIDAE	*Sitta*	x								x	x	x	x	x	

This material, provided by the author(s) as a supplement to Bird Migration Routes and Risk for Pathogen Dispersion into Western Mediterranean Wetlands, is not part of Emerging Infectious Diseases contents.

Family	Genus	A	B	C	D	E	F	G	H	I	J	K	L	M	N
TICHODROMADIDAE	*Tichodroma*	x								x	x	x	x	x	
CERTHIIDAE	*Certhia*	x								x	x	x	x	x	
REMIZIDAE	*Remiz*	x								x	x	x	x	x	
ORIOLIDAE	*Oriolus*	x								x	x	x	x	x	
LANIIDAE	*Lanius*	x								x	x	x	x	x	
CORVIDAE	*Corvus, Pica*	x								x	x	x	x	x	x
	Garrulus	x								x	x	x	x	x	
STURNIDAE	*Sturnus*	x								x	x	x	x	x	
PASSERIDAE	*Montifringilla, Petronia, Passer*	x								x	x	x	x	x	
FRINGILLIDAE	*Carduelis, Serinus, Fringilla,*	x								x	x	x	x	x	
EMBERIZIDAE	*Miliari, Plectrophenax, Emberiza*	x								x	x	x	x	x	
	Coccothraustes, Pyrrhula, Loxia	x								x	x	x	x	x	

*A: network of birdwatchers in the Camargue; B: monthly census on protected areas; C: Wetlands International census of waterbirds in mid January by plane and on ground; D: weekly census on Tour du Valat Estate (2,500 ha); E: count on roosting places during winter by a network of ornithologists; F: count on winter roosting places by plane monthly from September to March by a network of ornithologists; G: localization of colonies by plane and count on ground by Tour du Valat ornithologists in spring; H: counts of nests by plane; I: estimation of the number of breeding birds on Tour du Valat Estate every five years; J: weekly census by J. Blondel during three years based on visual contacts and songs on a transect; K: extrapolation according to habitat surfaces during the reproduction period; L: extrapolation according to habitat surfaces during migration and wintering; M: capture with mist nets during migration in the 1960s, 1970s, and since 2004; N: specific studies (detailed references provided on request).

This material, provided by the author(s) as a supplement to Bird Migration Routes and Risk for Pathogen Dispersion into Western Mediterranean Wetlands, is not part of Emerging Infectious Diseases contents.
