# Supplementary material for: Bird Migration Routes and Risk for Pathogen Dispersion into Western Mediterranean Wetlands
Source: Emerg Infect Dis. 2007 Mar;13(3):365–72. doi: 10.3201/eid1303.060301 (PMC2725901; doi:10.3201/eid1303.060301)
Supplement: Technical Appendix 2 — P. Bird migration routes and risk for pathogen dispersion into western Mediterranean wetlands. [file 06-0301_Techapp2-s2.pdf]

Id	Family	English Name	Latin Name	Areas from where birds come				
				Siberia Scandinavia	Continental Europe	Sub-Saharan Africa	J	F
1	GAVIIDAE	Red-throated Diver (Red-throated loon)	*Gavia stellata*	1			1	1
2	GAVIIDAE	Black-throated Diver (Arctic loon)	*Gavia arctica*	1			1	1
3	GAVIIDAE	Great Northern Diver (Common loon)	*Gavia immer*	1			1	1
4	PODICIPEDIDAE	Little Grebe	*Tachybaptus ruficollis*		1		1	1
5	PODICIPEDIDAE	Great crested Grebe	*Podiceps cristatus*		1		1	1
6	PODICIPEDIDAE	Red-necked Grebe	*Podiceps grisegena*	1			1	1
7	PODICIPEDIDAE	Slavonian Grebe	*Podiceps auritus*	1			1	1
8	PODICIPEDIDAE	Black-necked Grebe	*Podiceps nigricollis*		1		1	1
9	PROCELLARIIDAE	Cory's Shearwater	*Calonectris diomedea*				0	0
10	PROCELLARIIDAE	Yelkouan Shearwater	*Puffinus yelkouan*				1	1
11	HYDROBATIDAE	Storm Petrel	*Hydrobates pelagicus*				1	1
12	SULIDAE	Gannet	*Morus bassanus*				1	1
13	PHALACROCORACIDAE	Cormorant	*Phalacrocorax carbo*		1		1	1
14	ARDEIDAE	Bittern	*Botaurus stellaris*		1		1	1
15	ARDEIDAE	Little Bittern	*Ixobrychus minutus*		1	1	0	0
16	ARDEIDAE	Night Heron	*Nycticorax nycticorax*			1	1	1
17	ARDEIDAE	Squacco heron	*Ardeola ralloides*			1	0	0
18	ARDEIDAE	Cattle Egret	*Bubulcus ibis*				1	1
19	ARDEIDAE	Little Egret	*Egretta garzetta*				1	1
20	ARDEIDAE	Great White Egret	*Egretta alba*		1		1	1
21	ARDEIDAE	Grey Heron	*Ardea cinerea*				1	1
22	ARDEIDAE	Purple Heron	*Ardea purpurea*			1	0	0
23	CICONIIDAE	Black Stork	*Ciconia nigra*		1	1	1	1
24	CICONIIDAE	White Stork	*Ciconia ciconia*		1	1	1	1
25	THRESKIORNITHIDAE	Glossy Ibis	*Plegadis falcinellus*				1	1
26	THRESKIORNITHIDAE	Sacred Ibis	*Threskiornis aethiopicus*				1	1
27	THRESKIORNITHIDAE	Spoonbill	*Platalea leucorodia*		1		1	1
28	PHŒNICOPTERIDAE	Greater Flamingo	*Phoenicopterus ruber*			1	1	1
29	ANATIDAE	Mute Swan	*Cygnus olor*				1	1
30	ANATIDAE	Bewick's Swan	*Cygnus columbianus*	1			1	1
31	ANATIDAE	Bean Goose	*Anser fabalis*	1			1	1
32	ANATIDAE	Greylag Goose	*Anser anser*		1		1	1

33	ANATIDAE	Common Shelduck	*Tadorna tadorna*		1		1	1
34	ANATIDAE	Eurasian Wigeon	*Anas penelope*	1			1	1
35	ANATIDAE	Gadwall	*Anas strepera*		1		1	1
36	ANATIDAE	Common Teal (Green-winged Teal)	*Anas crecca*	1			1	1
37	ANATIDAE	Mallard	*Anas platyrhynchos*		1		1	1
38	ANATIDAE	Pintail (Northern Pintail)	*Anas acuta*	1		1	1	1
39	ANATIDAE	Garganey	*Anas querquedula*	1	1	1	0	1
40	ANATIDAE	Shoveler (Northern Shoveler)	*Anas clypeata*		1	1	1	1
41	ANATIDAE	Red-Crested Pochard	*Netta rufina*		1		1	1
42	ANATIDAE	Common Pochard	*Aythya ferina*	1			1	1
43	ANATIDAE	Ferruginous Duck	*Aythya nyroca*		1		1	1
44	ANATIDAE	Tufted Duck	*Aythya fuligula*	1			1	1
45	ANATIDAE	Scaup	*Aythya marila*	1			1	1
46	ANATIDAE	Eider	*Somateria mollissima*	1			1	1
47	ANATIDAE	Long-tailed Duck	*Clangula hyemalis*	1			1	1
48	ANATIDAE	Common Scoter	*Melanitta nigra*	1			1	1
49	ANATIDAE	Velvet Scoter	*Melanitta fusca*	1			1	1
50	ANATIDAE	Goldeneye	*Bucephala clangula*	1			1	1
51	ANATIDAE	Smew	*Mergus albellus*	1			1	0
52	ANATIDAE	Red-breasted Merganser	*Mergus serrator*	1			1	1
53	ANATIDAE	Goosander	*Mergus merganser*		1		1	1
54	ANATIDAE	White-fronted Goose	*Anser albifrons*	1			1	0
55	ACCIPITRIDAE	Honey Buzzard	*Pernis apivorus*		1	1	0	0
56	ACCIPITRIDAE	Black Kite	*Milvus migrans*		1	1	1	1
57	ACCIPITRIDAE	Red Kite	*Milvus milvus*		1		1	1
58	ACCIPITRIDAE	Egyptian Vulture	*Neophron percnopterus*			1	0	0
59	ACCIPITRIDAE	Short-toed Eagle	*Circaetus gallicus*			1	0	0
60	ACCIPITRIDAE	Marsh Harrier	*Circus aeruginosus*		1		1	1
61	ACCIPITRIDAE	Hen Harrier	*Circus cyaneus*		1		1	1
62	ACCIPITRIDAE	Montagu's Harrier	*Circus pygargus*		1	1	0	0
63	ACCIPITRIDAE	Goshawk	*Accipiter gentilis*		1		1	1
64	ACCIPITRIDAE	Sparrow Hawk	*Accipiter nisus*		1		1	1
65	ACCIPITRIDAE	Common Buzzard	*Buteo buteo*		1		1	1
66	ACCIPITRIDAE	Long-legged Buzzard	*Buteo rufinus*		1		1	1
67	ACCIPITRIDAE	Spotted Eagle	*Aquila clanga*		1		1	1
68	ACCIPITRIDAE	Booted Eagle	*Hieraaetus pennatus*		1	1	1	1

69	ACCIPITRIDAE	Bonelli's Eagle	*Hieraaetus fasciatus*				1	1
70	PANDIONIDAE	Osprey	*Pandion haliaetus*		1	1	1	1
71	FALCONIDAE	Lesser Kestrel	*Falco naumanni*			1	0	0
72	FALCONIDAE	Common Kestrel	*Falco tinnunculus*				1	1
73	FALCONIDAE	Red-footed Falcon	*Falco vespertinus*		1	1	0	0
74	FALCONIDAE	Merlin	*Falco columbarius*	1			1	1
75	FALCONIDAE	Hobby	*Falco subbuteo*		1	1	0	0
76	FALCONIDAE	Eleonora's Falcon	*Falco eleonorae*			1	0	0
77	FALCONIDAE	Peregrine Falcon	*Falco peregrinus*		1		1	1
78	PHASIANIDAE	Red-legged Partridge	*Alectoris rufa*				1	1
79	PHASIANIDAE	Quail	*Coturnix coturnix*		1	1	0	0
80	PHASIANIDAE	Common Pheasant	*Phasianus colchicus*				1	1
81	RALLIDAE	Water Rail	*Rallus aquaticus*		1		1	1
82	RALLIDAE	Corncrake	*Crex crex*		1	1	0	0
83	RALLIDAE	Spotted Crake	*Porzana porzana*		1	1	1	1
84	RALLIDAE	Little Crake	*Porzana parva*		1	1	0	0
85	RALLIDAE	Moorhen	*Gallinula chloropus*				1	1
86	RALLIDAE	Common Coot	*Fulica atra*		1		1	1
87	GRUIDAE	Common Crane	*Grus grus*	1			1	1
88	OTIDIDAE	Little Bustard	*Tetrax tetrax*				1	1
89	HAEMATOPODIDAE	Oystercatcher	*Haematopus ostralegus*				1	1
90	RECURVIROSTRIDAE	Black-winged Stilt	*Himantopus himantopus*			1	1	1
91	RECURVIROSTRIDAE	Avocet (Pied Avocet)	*Recurvirostra avosetta*				1	1
92	BURHINIDAE	Stone Curlew	*Burhinus oedicnemus*				1	1
93	GLAREOLIDAE	Pratincole	*Glareola pratincola*			1	0	0
94	CHARADRIIDAE	Little ringed Plover	*Charadrius dubius*		1	1	1	1
95	CHARADRIIDAE	Great ringed Plover	*Charadrius hiaticula*	1		1	1	1
96	CHARADRIIDAE	Kentish Plover	*Charadrius alexandrinus*				1	1
97	CHARADRIIDAE	Dotterel	*Charadrius morinellus*		1		0	0
98	CHARADRIIDAE	Golden Plover	*Pluvialis apricaria*	1			1	1
99	CHARADRIIDAE	Grey Plover	*Pluvialis squatarola*	1			1	1
100	CHARADRIIDAE	Lapwing	*Vanellus vanellus*		1		1	1
101	SCOLOPACIDAE	Black-tailed Godwit	*Limosa limosa*		1	1	1	1
102	SCOLOPACIDAE	Bar-tailed Godwit	*Limosa lapponica*	1		1	0	0
103	SCOLOPACIDAE	Terek Sandpiper	*Xenus cinereus*	1		1	0	0
104	SCOLOPACIDAE	Woodcock	*Scolopax rusticola*		1		1	1

105	SCOLOPACIDAE	Curlew Sandpiper	*Calidris ferruginea*	1		1	0	0
106	SCOLOPACIDAE	Temminck's Stint	*Calidris temminckii*	1		1	1	1
107	SCOLOPACIDAE	Broad-billed Sandpiper	*Limicola falcinellus*	1		1	0	0
108	SCOLOPACIDAE	Knot	*Calidris canutus*	1		1	1	1
109	SCOLOPACIDAE	Little Stint	*Calidris minuta*	1		1	1	1
110	SCOLOPACIDAE	Sanderling	*Calidris alba*	1		1	1	1
111	SCOLOPACIDAE	Dunlin	*Calidris alpina*	1		1	1	1
112	SCOLOPACIDAE	Snipe	*Gallinago gallinago*		1		1	1
113	SCOLOPACIDAE	Jack Snipe	*Lymnocryptes minimus*	1			1	1
114	SCOLOPACIDAE	Greenshank	*Tringa nebularia*	1		1	1	1
115	SCOLOPACIDAE	Spotted Redshank	*Tringa erythropus*	1		1	1	1
116	SCOLOPACIDAE	Green Sandpiper	*Tringa ochropus*	1		1	1	1
117	SCOLOPACIDAE	Common Redshank	*Tringa totanus*		1		1	1
118	SCOLOPACIDAE	Common Sandpiper	*Actitis hypoleucos*		1	1	1	1
119	SCOLOPACIDAE	Marsh Sandpiper	*Tringa stagnatilis*		1	1	0	0
120	SCOLOPACIDAE	Wood Sandpiper	*Tringa glareola*	1		1	0	0
121	SCOLOPACIDAE	Ruff	*Philomachus pugnax*	1		1	1	1
122	SCOLOPACIDAE	Curlew	*Numenius arquata*		1		1	1
123	SCOLOPACIDAE	Whimbrel	*Numenius phaeopus*	1		1	1	1
124	SCOLOPACIDAE	Red-necked Phalarope	*Phalaropus lobatus*	1		1	0	0
125	SCOLOPACIDAE	Turnstone	*Arenaria interpres*	1		1	1	1
126	STERCORARIIDAE	Great Skua	*Catharacta skua*				1	1
127	STERCORARIIDAE	Pomatorhine Skua	*Stercorarius pomarinus*				0	0
128	STERCORARIIDAE	Arctic Skua	*Stercorarius parasiticus*				1	1
129	LARIDAE	Hering Gull	*Larus argentatus*		1		1	1
130	LARIDAE	Lesser Black-backed Gull	*Larus fuscus*		1		1	1
131	LARIDAE	Common Gull	*Larus canus*		1		1	1
132	LARIDAE	Audoin's Gull	*Larus audouinii*				0	0
133	LARIDAE	Yellow-legged Herring Gull	*Larus cachinnans*				1	1
134	LARIDAE	Slender-billed Gull	*Larus genei*			1	1	1
135	LARIDAE	Mediterranean Gull	*Larus melanocephalus*			1	1	1
136	LARIDAE	Little Gull	*Larus minutus*		1	1	1	1
137	LARIDAE	Black-headed Gull	*Larus ridibundus*		1		1	1
138	STERNIDAE	Common Tern	*Sterna hirundo*			1	1	1
139	STERNIDAE	Little Tern	*Sterna albifrons*			1	0	0
140	STERNIDAE	Gull-billed Tern	*Gelochelidon nilotica*			1	0	0

141	STERNIDAE	Sandwich Tern	*Sterna sandvicensis*				1	1
142	STERNIDAE	Caspian Tern	*Sterna caspia*		1	1	0	0
143	STERNIDAE	Black Tern	*Chlidonias niger*		1	1	1	1
144	STERNIDAE	Whiskered Tern	*Chlidonias hybridus*		1	1	1	1
145	STERNIDAE	White-winged Black Tern	*Chlidonias leucopterus*		1	1	0	0
146	ALCIDAE	Razorbill	*Alca torda*				1	1
147	ALCIDAE	Puffin	*Fratercula arctica*				1	1
148	PTEROCLIDIDAE	Pin-tailed Sandgrouse	*Pterocles alchata*				1	1
149	COLUMBIDAE	Collared Dove	*Streptopelia decaocto*				1	1
150	COLUMBIDAE	Turtle Dove	*Streptopelia turtur*		1	1	0	0
151	COLUMBIDAE	Wood Pigeon	*Columba palumbus*		1		1	1
152	COLUMBIDAE	Stock Dove	*Columba oenas*		1		1	1
153	COLUMBIDAE	Rock Dove (Feral Pigeon)	*Columba livia*				1	1
154	CUCULIDAE	Common Cuckoo	*Cuculus canorus*		1	1	0	0
155	CUCULIDAE	Great Spotted Cuckoo	*Clamator glandarius*			1	0	0
156	TYTONIDAE	Barn Owl	*Tyto alba*				1	1
157	STRIGIDAE	Scops Owl	*Otus scops*			1	0	0
158	STRIGIDAE	Long-eared Owl	*Asio otus*		1		1	1
159	STRIGIDAE	Short-eared Owl	*Asio flammeus*		1		1	1
160	STRIGIDAE	Eagle Owl	*Bubo bubo*				1	1
161	STRIGIDAE	Tawny Owl	*Strix aluco*				1	1
162	STRIGIDAE	Little Owl	*Athene noctua*				1	1
163	CAPRIMULGIDAE	Nightjar	*Caprimulgus europaeus*			1	0	0
164	APODIDAE	Common Swift	*Apus apus*		1	1	0	0
165	APODIDAE	Pallid Swift	*Apus pallidus*			1	0	0
166	APODIDAE	Alpine Swift	*Apus melba*			1	0	0
167	ALCEDINIDAE	European Kingfisher	*Alcedo atthis*		1		1	1
168	MEROPIDAE	European Bee-eater	*Merops apiaster*			1	0	0
169	CORACIIDAE	European Roller	*Coracias garrulus*			1	0	0
170	UPUPIDAE	Hoopoe	*Upupa epops*			1	0	1
171	PICIDAE	Wryneck	*Jynx torquilla*		1	1	1	1
172	PICIDAE	Green Woodpecker	*Picus viridis*				1	1
173	PICIDAE	Lesser Spotted Woodpecker	*Dendrocopos minor*				1	1
174	PICIDAE	Greater Spotted Woodpecker	*Dendrocopos major*				1	1
175	ALAUDIDAE	Crested Lark	*Galerida cristata*				1	1
176	ALAUDIDAE	Wood Lark	*Lullula arborea*		1		1	1

177	ALAUDIDAE	Skylark	*Alauda arvensis*		1		1	1
178	ALAUDIDAE	Short-toed Lark	*Calandrella brachydactyla*				0	0
179	ALAUDIDAE	Calandra Lark	*Melanocorypha calandra*				1	1
180	HIRUNDINIDAE	Sand Martin	*Riparia riparia*		1	1	0	0
181	HIRUNDINIDAE	Crag Martin	*Hirundo rupestris*				1	1
182	HIRUNDINIDAE	Barn Swallow	*Hirundo rustica*		1	1	1	1
183	HIRUNDINIDAE	Red-rumped Swallow	*Hirundo daurica*			1	0	0
184	HIRUNDINIDAE	House Martin	*Delichon urbica*		1	1	0	1
185	MOTACILLIDAE	Water Pipit	*Anthus spinoletta*		1		1	1
186	MOTACILLIDAE	Tawny Pipit	*Anthus campestris*			1	0	0
187	MOTACILLIDAE	Meadow Pipit	*Anthus pratensis*		1		1	1
188	MOTACILLIDAE	Tree Pipit	*Anthus trivialis*		1	1	0	0
189	MOTACILLIDAE	Red-throated Pipit	*Anthus cervinus*	1		1	0	0
190	MOTACILLIDAE	Yellow Wagtail (Blue-headed Wagtail)	*Motacilla flava*		1	1	1	1
191	MOTACILLIDAE	White Wagtail (Pied Wagtail)	*Motacilla alba*		1		1	1
192	MOTACILLIDAE	Grey Wagtail	*Motacilla cinerea*		1		1	1
193	CINCLIDAE	Dipper	*Cinclus cinclus*				0	0
194	TROGLODYTIDAE	Wren	*Troglodytes troglodytes*		1		1	1
195	PRUNELLIDAE	Dunnock (Hedge Sparrow)	*Prunella modularis*		1		1	1
196	PRUNELLIDAE	Alpine Accentor	*Prunella collaris*		1		1	1
197	TURDIDAE	Black-eared Wheatear	*Oenanthe hispanica*			1	0	0
198	TURDIDAE	Wheatear (Northern Wheatear)	*Oenanthe oenanthe*		1	1	0	0
199	TURDIDAE	Stonechat	*Saxicola torquata*		1		1	1
200	TURDIDAE	Whinchat	*Saxicola rubetra*		1	1	0	0
201	TURDIDAE	Black Redstart	*Phoenicurus ochruros*		1	1	1	1
202	TURDIDAE	Common Redstart	*Phoenicurus phoenicurus*		1	1	0	0
203	TURDIDAE	Eurasian Robin	*Erithacus rubecula*		1		1	1
204	TURDIDAE	Common Nightingale	*Luscinia megarhynchos*		1	1	0	0
205	TURDIDAE	European Blackbird	*Turdus merula*		1		1	1
206	TURDIDAE	Rock Trush	*Monticola saxatilis*			1	0	0
207	TURDIDAE	Blue Rock Trush	*Monticola solitarius*				1	1
208	TURDIDAE	Ring Ouzel	*Turdus torquatus*				0	1
209	TURDIDAE	Song Trush	*Turdus philomelos*		1		1	1
210	TURDIDAE	Redwing	*Turdus iliacus*	1			1	1
211	TURDIDAE	Fieldfare	*Turdus pilaris*		1		1	1
212	TURDIDAE	Mistle Trush	*Turdus viscivorus*		1		1	1

213	TURDIDAE	Bluethroat	*Luscinia svecica*				1	1
214	SYLVIIDAE	Marsh Warbler	*Acrocephalus palustris*		1	1	0	0
215	SYLVIIDAE	Great Reed Warbler	*Acrocephalus arundinaceus*		1	1	0	0
216	SYLVIIDAE	Reed Warbler	*Acrocephalus scirpaceus*		1	1	0	0
217	SYLVIIDAE	Firecrest	*Regulus ignicapillus*		1		1	1
218	SYLVIIDAE	Goldcrest	*Regulus regulus*		1		1	1
219	SYLVIIDAE	Chiffchaff	*Phylloscopus collybita*		1		1	1
220	SYLVIIDAE	Wood Warbler	*Phylloscopus sibilatrix*		1	1	0	0
221	SYLVIIDAE	Willow Warbler	*Phylloscopus trochilus*		1	1	0	0
222	SYLVIIDAE	Bonelli's Warbler	*Phylloscopus bonelli*		1	1	0	0
223	SYLVIIDAE	Sedge Warbler	*Acrocephalus schoenobaenus*		1	1	0	0
224	SYLVIIDAE	Aquatic Warbler	*Acrocephalus paludicola*		1	1	0	0
225	SYLVIIDAE	Moustached Warbler	*Acrocephalus melanopogon*				1	1
226	SYLVIIDAE	Grasshopper Warbler	*Locustella naevia*		1	1	0	0
227	SYLVIIDAE	Savi's Warbler	*Locustella luscinioides*			1	0	0
228	SYLVIIDAE	Melodious Warbler	*Hippolais polyglotta*			1	0	0
229	SYLVIIDAE	Icterine Warbler	*Hippolais icterina*		1	1	0	0
230	SYLVIIDAE	Dartford Warbler	*Sylvia undata*				1	1
231	SYLVIIDAE	Subalpine Warbler	*Sylvia cantillans*			1	1	1
232	SYLVIIDAE	Orphean Warbler	*Sylvia hortensis*			1	0	0
233	SYLVIIDAE	Sardinian Warbler	*Sylvia melanocephala*				1	1
234	SYLVIIDAE	Whitethroat	*Sylvia communis*		1	1	0	0
235	SYLVIIDAE	Garden Warbler	*Sylvia borin*		1	1	0	0
236	SYLVIIDAE	Lesser Whitethroat	*Sylvia curruca*		1	1	0	0
237	SYLVIIDAE	Blackcap	*Sylvia atricapilla*		1		1	1
238	SYLVIIDAE	Spectacled Warbler	*Sylvia conspicillata*				1	1
239	SYLVIIDAE	Fan-tailed Warbler	*Cisticola juncidis*				1	1
240	SYLVIIDAE	Cetti's Warbler	*Cettia cetti*				1	1
241	MUSCICAPIDAE	Pied Flycatcher	*Ficedula hypoleuca*		1	1	0	0
242	MUSCICAPIDAE	Spotted Flycatcher	*Muscicapa striata*		1	1	0	0
243	TIMALIIDAE	Bearded Tit	*Panurus biarmicus*				1	1
244	AEGITHALIDAE	Long-tailed Tit	*Aegithalos caudatus*				1	1
245	PARIDAE	Coal Tit	*Parus ater*		1		1	1
246	PARIDAE	Great Tit	*Parus major*				1	1
247	PARIDAE	Blue Tit	*Parus caeruleus*				1	1
248	SITTIDAE	Nuthatch	*Sitta europaea*				1	1

249	TICHODROMADIDAE	Wall Creeper	*Tichodroma muraria*		1		1	1
250	CERTHIIDAE	Short-toed Treecreeper	*Certhia brachydactyla*				1	1
251	REMIZIDAE	Penduline Tit	*Remiz pendulinus*		1		1	1
252	ORIOLIDAE	Golden Oriole	*Oriolus oriolus*			1	0	0
253	LANIIDAE	Red-backed Shrike	*Lanius collurio*		1	1	0	0
254	LANIIDAE	Lesser Grey Shrike	*Lanius minor*			1	0	0
255	LANIIDAE	Great Gray Shrike	*Lanius excubitor*		1	1	1	1
256	LANIIDAE	Mediterranean Great Gray Shrike	*Lanius meridionalis*				1	1
257	LANIIDAE	Woodchat Shrike	*Lanius senator*		1	1	0	0
258	CORVIDAE	Common Magpie	*Pica pica*				1	1
259	CORVIDAE	Raven	*Corvus corax*				1	1
260	CORVIDAE	Eurasian Jay	*Garrulus glandarius*				1	1
261	CORVIDAE	Carrion Crow	*Corvus corone*		1		1	1
262	CORVIDAE	Rook	*Corvus frugilegus*		1		1	1
263	CORVIDAE	Jackdaw	*Corvus monedula*				1	1
264	STURNIDAE	Spotless Starling	*Sturnus unicolor*				1	1
265	STURNIDAE	Common Starling	*Sturnus vulgaris*		1		1	1
266	PASSERIDAE	Snow Finch	*Montifringilla nivalis*		1		1	1
267	PASSERIDAE	Rock Sparrow	*Petronia petronia*				0	0
268	PASSERIDAE	Tree Sparrow	*Passer montanus*				1	1
269	PASSERIDAE	House Sparrow	*Passer domesticus*				1	1
270	FRINGILLIDAE	Greenfinch	*Carduelis chloris*		1		1	1
271	FRINGILLIDAE	Citril Finch	*Serinus citrinella*		1		1	1
272	FRINGILLIDAE	Siskin	*Carduelis spinus*	1			1	1
273	FRINGILLIDAE	Redpoll	*Carduelis flammea*	1			1	1
274	FRINGILLIDAE	European Serin	*Serinus serinus*		1		1	1
275	FRINGILLIDAE	Brambling	*Fringilla montifringilla*	1			1	1
276	FRINGILLIDAE	Chaffinch	*Fringilla coelebs*		1		1	1
277	FRINGILLIDAE	Linnet	*Carduelis cannabina*		1		1	1
278	FRINGILLIDAE	Hawfinch	*Coccothraustes coccothraustes*		1		1	1
279	FRINGILLIDAE	Goldfinch	*Carduelis carduelis*		1		1	1
280	FRINGILLIDAE	Bullfinch	*Pyrrhula pyrrhula*		1		1	0
281	FRINGILLIDAE	Crossbill	*Loxia curvirostra*	1			1	1
282	EMBERIZIDAE	Cirl Bunting	*Emberiza cirlus*		1		1	1
283	EMBERIZIDAE	Corn Bunting	*Miliaria calandra*		1		1	1
284	EMBERIZIDAE	Ortolan Bunting	*Emberiza hortulana*			1	0	0

285	EMBERIZIDAE	Yellowhammer	*Emberiza citrinella*		1		1	1
286	EMBERIZIDAE	Rock Bunting	*Emberiza cia*				1	1
287	EMBERIZIDAE	Reed Bunting	*Emberiza schoeniclus*		1		1	1
288	EMBERIZIDAE	Snow Bunting	*Plectrophenax nivalis*	1			1	1
289	EMBERIZIDAE	Pine Bunting	*Emberiza leucocephalos*	1			1	1

Bird diversity										Bird abundance											
M	A	M	J	J	A	S	O	N	D	J	F	M	A	M	J	J	A	S	O	N	D
1	1	0	0	0	0	0	1	1	1	1	1	1	1	0	0	0	0	0	1	1	1
1	1	0	0	0	0	0	1	1	1	1	1	1	1	0	0	0	0	0	1	1	1
1	1	0	0	0	0	0	1	1	1	1	1	1	1	0	0	0	0	0	1	1	1
1	1	1	1	1	1	1	1	1	1	100	100	100	100	100	100	100	100	100	100	100	100
1	1	1	1	1	1	1	1	1	1	1000	1000	1000	1000	100	100	100	100	100	1000	1000	1000
1	1	0	0	0	0	0	1	1	1	1	1	1	1	0	0	0	0	0	1	1	1
1	1	0	0	0	0	0	1	1	1	1	1	1	1	0	0	0	0	0	1	1	1
1	1	1	1	1	1	1	1	1	1	1000	1000	100	100	1	1	1	1000	1000	1000	1000	1000
1	1	1	1	1	1	1	0	0	0	0	0	1	1	1	1	1	1	1	0	0	0
1	1	1	1	1	1	1	1	1	1	1000	1000	1000	1000	100	100	100	100	100	100	1000	1000
1	1	1	1	1	1	1	1	1	1	1	1	1	1	1	1	1	1	1	1	1	1
1	1	1	1	1	1	1	1	1	1	10	10	10	10	10	10	10	10	10	100	100	10
1	1	1	1	1	1	1	1	1	1	1000	1000	1000	10	1	1	1	10	10	1000	1000	1000
1	1	1	1	1	1	1	1	1	1	10	10	10	10	10	10	10	10	10	10	10	10
0	0	1	1	1	1	0	0	0	0	0	0	0	0	10	10	10	10	0	0	0	0
1	1	1	1	1	1	1	1	1	1	1	1	100	100	100	100	100	100	100	100	1	1
0	0	1	1	1	1	1	0	0	0	0	0	0	0	100	100	100	100	100	0	0	0
1	1	1	1	1	1	1	1	1	1	1000	1000	1000	1000	1000	1000	1000	1000	1000	1000	1000	1000
1	1	1	1	1	1	1	1	1	1	1000	1000	1000	1000	1000	1000	1000	1000	1000	1000	1000	1000
1	1	1	1	1	1	1	1	1	1	100	100	100	1	1	1	1	1	100	100	100	100
1	1	1	1	1	1	1	1	1	1	1000	1000	1000	1000	1000	1000	1000	1000	1000	1000	1000	1000
1	1	1	1	1	1	1	1	0	0	0	0	100	1000	1000	1000	1000	1000	1000	10	0	0
1	0	0	0	0	1	1	1	1	1	1	1	1	0	0	0	0	10	10	1	1	1
1	1	1	1	1	1	1	1	1	1	1	1	1	10	1	1	1	10	10	10	10	1
1	1	1	1	1	1	1	1	1	1	1	1	1	1	1	1	1	1	1	1	1	1
1	1	1	1	1	1	1	1	1	1	1	1	1	1	1	1	1	1	1	1	1	1
1	1	1	1	1	1	1	1	1	1	1	1	1	10	10	10	10	10	10	1	1	1
1	1	1	1	1	1	1	1	1	1	1000	1000	10000	10000	10000	10000	10000	10000	10000	10000	1000	1000
1	1	1	1	1	1	1	1	1	1	100	100	100	100	10	10	10	100	100	100	100	100
1	0	0	0	0	0	0	1	1	1	10	10	10	0	0	0	0	0	0	10	10	10
0	0	0	0	0	0	0	0	1	1	1	1	0	0	0	0	0	0	0	0	1	1
1	1	1	1	1	1	1	1	1	1	100	100	1	1	1	1	1	1	1	100	100	100

1	1	1	1	1	1	1	1	1	1	100	1000	1000	100	100	100	100	1	100	1000	1000	100
1	1	0	0	0	1	1	1	1	1	1000	1000	1000	10	0	0	0	10	100	1000	1000	1000
1	1	1	1	1	1	1	1	1	1	1000	1000	1000	1	1	1	1	10	1000	1000	1000	1000
1	1	1	1	1	1	1	1	1	1	10000	1000	1000	10	1	1	100	1000	1000	10000	10000	10000
1	1	1	1	1	1	1	1	1	1	10000	1000	1000	1000	1000	1000	1000	10000	10000	10000	10000	10000
1	1	0	0	0	1	1	1	1	1	1000	1000	100	10	0	0	0	10	100	100	1000	1000
1	1	1	1	1	1	1	1	0	0	0	10	1000	1000	10	1	1	1000	1000	10	0	0
1	1	1	1	1	1	1	1	1	1	1000	1000	1000	1	1	1	1	100	1000	1000	1000	1000
1	1	1	1	1	1	1	1	1	1	1000	1000	100	100	100	100	100	100	100	100	1000	1000
1	1	1	1	1	1	1	1	1	1	1000	1000	100	1	1	1	1	1	1000	10000	10000	1000
0	0	0	0	0	0	0	1	1	1	1	1	0	0	0	0	0	0	0	1	1	1
1	1	0	0	0	0	1	1	1	1	1000	1000	100	1	0	0	0	0	10	10	100	1000
1	1	0	0	0	0	0	0	1	1	1	1	1	1	0	0	0	0	0	0	1	1
1	1	1	1	1	1	1	1	1	1	10	10	10	1	1	1	1	1	1	1	10	10
1	0	0	0	0	0	0	0	1	1	1	1	1	0	0	0	0	0	0	0	1	1
1	0	0	0	0	0	0	0	1	1	10	10	10	0	0	0	0	0	0	0	10	10
1	0	0	0	0	0	0	0	1	1	100	100	100	0	0	0	0	0	0	0	100	100
1	0	0	0	0	0	0	0	1	1	1	10	10	0	0	0	0	0	0	0	1	1
0	0	0	0	0	0	0	0	1	1	1	0	0	0	0	0	0	0	0	0	1	1
1	1	0	0	0	0	0	0	1	1	10	10	10	10	0	0	0	0	0	0	10	10
0	0	0	0	0	0	0	0	0	1	1	1	0	0	0	0	0	0	0	0	0	1
0	0	0	0	0	0	0	0	1	1	1	0	0	0	0	0	0	0	0	0	1	1
0	1	1	1	1	1	1	0	0	0	0	0	0	1000	1000	1	1	1000	1000	0	0	0
1	1	1	1	1	1	1	1	1	1	1	1	1000	1000	10	10	10	1000	1000	1	1	1
1	1	0	0	0	0	1	1	1	1	100	100	100	10	0	0	0	0	100	100	100	100
1	1	1	1	1	1	1	0	0	0	0	0	1	1	1	1	1	1	1	0	0	0
1	1	1	1	1	1	1	1	0	0	0	0	1	10	10	10	10	10	10	10	0	0
1	1	1	1	1	1	1	1	1	1	100	100	100	10	10	10	10	10	100	100	100	100
1	1	0	0	0	0	1	1	1	1	10	10	100	10	0	0	0	0	1	100	100	10
0	1	1	1	1	1	1	1	0	0	0	0	0	10	10	10	10	10	10	1	0	0
1	0	0	0	0	0	0	1	1	1	1	1	1	0	0	0	0	0	0	1	1	1
1	1	1	1	1	1	1	1	1	1	10	10	10	10	10	10	10	100	100	100	10	10
1	1	1	1	1	1	1	1	1	1	100	100	100	100	10	10	10	100	100	100	100	100
1	1	0	0	0	0	1	1	1	1	1	1	1	1	0	0	0	0	1	1	1	1
1	0	0	0	0	0	0	1	1	1	1	1	1	0	0	0	0	0	0	1	1	1
1	1	1	1	1	1	1	1	1	1	1	1	1	1	1	1	1	1	1	1	1	1

1	1	1	1	1	1	1	1	1	1	1	1	1	1	1	1	1	1	1	1	1	1
1	1	1	1	1	1	1	1	1	1	1	1	1	1	1	1	1	10	10	10	1	1
1	1	1	1	1	1	1	0	0	0	0	0	10	10	10	10	10	10	10	0	0	0
1	1	1	1	1	1	1	1	1	1	100	100	100	100	100	100	100	100	100	100	100	100
0	1	1	1	1	1	1	0	0	0	0	0	0	10	10	1	1	1	1	0	0	0
1	0	0	0	0	0	0	1	1	1	100	100	100	0	0	0	0	0	0	100	100	100
1	1	1	1	1	1	1	1	1	0	0	0	10	100	100	100	10	100	100	100	1	0
0	0	1	1	1	1	1	0	0	0	0	0	0	0	1	1	1	1	1	0	0	0
1	1	1	1	1	1	1	1	1	1	10	10	10	1	1	1	1	1	1	10	10	10
1	1	1	1	1	1	1	1	1	1	1000	1000	1000	1000	1000	1000	1000	1000	1000	1000	1000	1000
0	1	1	1	1	1	1	1	0	0	0	0	0	10	10	1	1	1	10	10	0	0
1	1	1	1	1	1	1	1	1	1	1000	1000	100	100	100	100	100	1000	1000	1000	1000	1000
1	1	1	1	1	1	1	1	1	1	1000	1000	1000	1000	1000	1000	1000	1000	1000	1000	1000	1000
0	1	0	0	0	0	1	1	0	0	0	0	0	1	0	0	0	0	1	1	0	0
1	1	0	0	1	1	1	1	1	1	1	1	10	10	0	0	1	1	10	10	1	1
1	1	0	0	1	1	0	0	0	0	0	0	1	1	0	0	1	1	0	0	0	0
1	1	1	1	1	1	1	1	1	1	1000	1000	1000	1000	1000	1000	1000	1000	1000	1000	1000	1000
1	1	1	1	1	1	1	1	1	1	10000	1000	1000	1000	1000	1000	1000	1000	10000	10000	10000	10000
1	0	0	0	0	0	0	1	1	1	10	100	10	0	0	0	0	0	0	10	100	10
1	1	1	1	1	1	1	1	1	1	100	100	100	100	100	100	100	100	100	100	100	100
1	1	1	1	1	1	1	1	1	1	1	1	10	10	10	10	10	10	1	1	1	1
1	1	1	1	1	1	1	1	1	1	1	1	10	100	100	100	100	100	10	1	1	1
1	1	1	1	1	1	1	1	1	1	100	100	100	100	100	100	100	100	100	100	100	100
1	1	1	1	1	1	1	1	1	1	1	1	10	10	10	10	10	10	10	10	1	1
0	1	1	1	1	1	1	0	0	0	0	0	0	10	10	10	10	10	10	0	0	0
1	1	1	1	1	1	1	1	1	1	1	1	1000	1000	100	1	1000	1000	1000	100	1	1
1	1	1	1	1	1	1	1	1	1	10	10	100	1000	1000	10	10	1000	1000	1000	10	10
1	1	1	1	1	1	1	1	1	1	100	100	100	100	100	100	100	1000	1000	100	100	100
0	1	1	0	0	1	1	1	0	0	0	0	0	1	1	0	0	10	10	10	0	0
1	1	0	0	0	1	1	1	1	1	100	100	100	1	0	0	0	10	10	10	100	100
1	1	1	1	1	1	1	1	1	1	100	100	100	1000	100	100	100	100	1000	100	100	100
1	1	1	1	1	1	1	1	1	1	1000	1000	1000	10	10	10	10	10	10	1000	1000	1000
1	1	1	1	1	1	1	1	1	1	1	1000	10000	1000	10	10	10000	10000	100	10	1	1
1	1	1	1	1	1	1	1	1	0	0	0	1	10	10	1	1	1	10	10	1	0
1	1	1	1	1	1	1	0	0	0	0	0	1	1	1	1	1	1	1	0	0	0
1	0	0	0	0	0	0	1	1	1	10	10	10	0	0	0	0	0	0	10	10	10

0	1	1	1	1	1	1	1	0	0	0	0	0	1000	1000	10	1000	1000	1000	10	0	0
1	1	1	0	1	1	1	1	1	1	1	1	1	10	10	0	10	100	100	10	1	1
0	0	1	0	1	1	1	0	0	0	0	0	0	0	1	0	1	1	1	0	0	0
1	1	1	1	1	1	1	1	1	1	1	1	1	10	10	10	1	10	10	1	1	1
1	1	1	1	1	1	1	1	1	1	100	100	100	100	1000	10	10	10000	10000	1000	1000	100
1	1	1	1	1	1	1	1	1	1	10	10	10	1000	1000	100	10	10	10	10	10	10
1	1	1	1	1	1	1	1	1	1	1000	1000	10000	10000	1000	100	100	100	1000	10000	1000	1000
1	1	1	0	0	1	1	1	1	1	10000	10000	10000	100	1	0	0	1	10000	10000	10000	10000
1	0	0	0	0	0	0	1	1	1	100	100	100	0	0	0	0	0	0	100	100	100
1	1	1	1	1	1	1	1	1	1	1	1	10	1000	1000	10	100	1000	1000	100	10	1
1	1	1	1	1	1	1	1	1	1	10	10	10	1000	1000	1	100	1000	1000	100	10	10
1	1	1	1	1	1	1	1	1	1	10	10	10	100	10	100	1000	1000	100	100	10	10
1	1	1	1	1	1	1	1	1	1	100	100	100	100	100	100	100	100	100	100	100	100
1	1	1	1	1	1	1	1	1	1	1	1	1	100	100	1	1000	1000	100	1	1	1
1	1	1	1	1	1	1	0	0	0	0	0	10	10	10	10	10	10	10	0	0	0
1	1	1	1	1	1	1	1	1	0	0	0	10	1000	100	10	1000	1000	1000	100	1	0
1	1	1	1	1	1	1	1	1	1	10	100	1000	10000	1000	1	10	100	100	100	10	10
1	1	1	1	1	1	1	1	1	1	100	100	100	10	10	10	10	100	100	100	100	100
1	1	1	1	1	1	1	1	1	1	1	1	1	100	100	1	1	100	100	1	1	1
1	1	1	1	1	1	1	0	0	0	0	0	1	1	1	1	1	1	1	0	0	0
1	1	1	1	1	1	1	1	1	1	1	1	10	10	10	1	1	10	10	1	1	1
1	1	1	1	1	1	1	1	1	1	1	1	1	1	1	1	1	1	1	1	1	1
0	0	1	1	0	1	1	0	0	0	0	0	0	0	1	1	0	1	1	0	0	0
1	1	1	0	0	1	1	1	1	1	1	1	1	10	10	0	0	10	10	10	1	1
1	0	0	0	0	0	1	1	1	1	1	1	1	0	0	0	0	0	1	1	1	1
1	1	1	1	1	1	1	1	1	1	10	10	10	10	1	1	1	1	1	1	10	10
1	1	1	1	1	1	1	1	1	1	10	10	10	1	1	1	1	1	1	1	1	10
0	1	1	1	1	1	1	0	0	0	0	0	0	1	1	1	1	1	1	0	0	0
1	1	1	1	1	1	1	1	1	1	10000	10000	10000	10000	10000	10000	10000	10000	10000	10000	10000	10000
1	1	1	1	1	1	1	1	1	1	10	10	10	100	100	100	100	100	10	10	10	10
1	1	1	1	1	1	1	1	1	1	100	100	1000	1000	1000	1000	1000	1000	100	100	100	100
1	1	1	1	1	1	1	1	1	1	1	1	100	100	100	1	1	100	100	1	1	1
1	1	1	1	1	1	1	1	1	1	10000	10000	10000	1000	1000	1000	1000	1000	1000	10000	10000	10000
1	1	1	1	1	1	1	1	1	1	1	1	1	100	100	100	100	100	100	1	1	1
0	1	1	1	1	1	1	0	0	0	0	0	0	100	100	100	100	100	100	0	0	0
1	1	1	1	1	1	1	0	0	0	0	0	10	100	100	100	100	100	10	0	0	0

This material, provided by the authors as a supplement to the article, is not part of Emerging Infectious Diseases contents.

1	1	1	1	1	1	1	1	1	1	100	100	100	100	100	100	100	100	100	1000	100	100
1	1	1	1	1	1	1	1	1	0	0	0	1	1	1	1	1	1	10	10	1	0
1	1	1	1	1	1	1	1	1	1	1	1	1	1000	1000	10	1000	1000	1	1	1	1
1	1	1	1	1	1	1	1	1	1	10	10	10	100	100	10	100	100	10	10	10	10
0	1	1	1	1	1	1	0	0	0	0	0	0	10	10	10	1	10	10	0	0	0
1	1	1	1	1	1	1	1	1	1	100	100	100	10	1	1	1	1	1	100	100	100
0	0	0	0	0	0	0	0	1	1	1	1	0	0	0	0	0	0	0	0	1	1
1	1	1	1	1	1	1	1	1	1	1000	1000	1000	1000	1000	1000	1000	1000	1000	1000	1000	1000
1	1	1	1	1	1	1	1	1	1	100	100	100	100	100	100	100	100	100	100	100	100
0	1	1	1	1	1	1	1	0	0	0	0	0	100	100	100	100	100	100	1	0	0
1	1	1	1	1	1	1	1	1	1	1000	1000	1000	100	100	100	100	100	1000	10000	10000	1000
1	0	0	0	0	0	1	1	1	1	10	100	10	0	0	0	0	0	100	100	10	10
1	1	1	1	1	1	1	1	1	1	1000	1000	1000	1000	1000	1000	1000	1000	1000	1000	1000	1000
1	1	1	1	1	1	1	0	0	0	0	0	10	100	100	100	100	100	100	0	0	0
1	1	1	1	1	1	1	0	0	0	0	0	10	10	10	10	10	10	10	0	0	0
1	1	1	1	1	1	1	1	1	1	10	10	10	10	10	10	10	10	10	10	10	10
1	1	1	1	1	1	1	1	0	0	0	0	1	10	10	10	10	10	10	10	0	0
1	1	1	1	1	1	1	1	1	1	10	10	10	10	10	10	10	10	10	10	10	10
1	0	0	0	0	0	0	1	1	1	1	1	1	0	0	0	0	0	0	1	1	1
1	1	1	1	1	1	1	1	1	1	10	10	10	10	10	10	10	10	10	10	10	10
1	1	1	1	1	1	1	1	1	1	10	10	10	10	10	10	10	10	10	10	10	10
1	1	1	1	1	1	1	1	1	1	10	10	10	10	10	10	10	10	10	10	10	10
0	1	1	1	1	1	1	0	0	0	0	0	0	10	10	10	10	10	10	0	0	0
1	1	1	1	1	1	1	1	0	0	0	0	10	1000	10000	1000	10000	10000	1000	1	0	0
1	1	1	1	1	1	1	0	0	0	0	0	10	10	10	10	10	10	10	0	0	0
1	1	1	1	1	1	1	1	0	0	0	0	10	100	100	100	100	100	100	1	0	0
1	1	1	1	1	1	1	1	1	1	10	10	10	10	10	100	100	100	100	100	10	10
0	1	1	1	1	1	1	0	0	0	0	0	0	100	100	100	100	100	100	0	0	0
0	1	1	1	1	1	1	1	0	0	0	0	0	10	10	10	10	10	10	1	0	0
1	1	1	1	1	1	1	0	0	0	0	1	100	100	100	100	100	100	100	0	0	0
1	1	0	0	0	1	1	1	1	1	1	1	10	10	0	0	0	10	10	1	1	1
1	1	1	1	1	1	1	1	1	1	100	100	100	100	100	100	100	100	100	100	100	100
1	1	1	1	1	1	1	1	1	1	10	10	10	10	10	10	10	10	10	10	10	10
1	1	1	1	1	1	1	1	1	1	100	100	100	100	100	100	100	100	100	100	100	100
1	1	1	1	1	1	1	1	1	1	10	10	10	10	10	10	10	10	10	10	10	10
1	1	1	1	1	1	1	1	1	1	10	10	10	10	10	10	10	10	10	10	10	10

1	1	1	1	1	1	1	1	1	1	10000	10000	10000	100	100	100	100	100	100	10000	10000	10000
1	1	1	1	1	1	1	0	0	0	0	0	10	10	10	10	10	10	10	0	0	0
1	1	1	1	1	1	1	1	1	1	10	10	10	10	10	10	10	10	10	10	10	10
1	1	1	1	1	1	1	1	0	0	0	0	100	10000	10000	10	10000	10000	10000	10	0	0
1	1	1	1	1	1	1	1	1	1	10	10	10	10	10	10	10	10	10	10	10	10
1	1	1	1	1	1	1	1	1	1	1	1	100	10000	10000	1000	10000	10000	10000	1000	10	1
1	1	1	1	1	1	1	0	0	0	0	0	1	10	10	10	10	10	10	0	0	0
1	1	1	1	1	1	1	1	0	0	0	1	100	1000	1000	1000	1000	1000	1000	100	0	0
0	0	0	0	0	0	0	1	1	1	1000	1000	0	0	0	0	0	0	0	1000	1000	1000
1	1	1	1	1	1	1	1	0	0	0	0	1	100	100	100	100	100	100	10	0	0
1	1	0	0	0	0	1	1	1	1	10000	10000	1000	1	0	0	0	0	10	1000	10000	10000
1	1	1	0	0	1	1	1	1	0	0	0	10	100	100	0	0	100	1000	1000	1	0
0	1	1	0	0	0	1	0	0	0	0	0	0	10	10	0	0	0	10	0	0	0
1	1	1	1	1	1	1	1	1	1	1	1	100	10000	10000	1000	1000	10000	10000	100	1	1
1	1	1	1	1	1	1	1	1	1	100	1000	1000	10	10	10	10	10	10	1000	1000	100
1	0	0	0	0	0	1	1	1	1	10	10	10	0	0	0	0	0	10	10	10	1
0	0	0	0	0	0	0	0	0	0	0	0	0	0	0	0	0	0	0	0	0	0
1	1	1	1	1	1	1	1	1	1	1000	1000	1000	10	1	1	1	1	100	10000	10000	1000
1	1	1	0	0	0	1	1	1	1	1000	1000	10000	100	10	0	0	0	100	10000	10000	1000
1	1	0	0	0	0	0	1	1	1	10	10	10	1	0	0	0	0	0	10	10	10
1	1	1	1	1	1	1	0	0	0	0	0	10	10	10	10	10	10	10	0	0	0
1	1	1	0	0	1	1	1	0	0	0	0	100	1000	1000	0	0	1000	1000	100	0	0
1	1	1	1	1	1	1	1	1	1	10	100	100	10	10	10	10	10	100	100	10	10
1	1	1	0	0	1	1	1	0	0	0	0	1	100	100	0	0	100	100	100	0	0
1	1	1	1	1	1	1	1	1	1	100	100	1000	1000	100	100	100	100	100	1000	100	100
1	1	1	1	1	1	1	1	1	0	0	0	100	1000	1000	10	10	1000	1000	1000	10	0
1	1	1	1	1	1	1	1	1	1	1000	1000	10000	1000	100	100	100	100	1000	10000	1000	1000
1	1	1	1	1	1	1	1	0	0	0	0	1	10000	10000	10000	10000	10000	10000	10	0	0
1	1	1	1	1	1	1	1	1	1	10000	10000	10000	100	100	100	100	100	1000	10000	10000	10000
0	1	1	1	1	1	1	0	0	0	0	0	0	100	100	100	100	100	100	0	0	0
1	1	1	1	1	1	1	1	1	1	100	100	100	100	100	100	100	100	100	100	100	100
1	1	1	0	0	0	1	1	1	1	0	100	100	100	1	0	0	0	100	100	100	1
1	1	1	1	1	1	1	1	1	1	1000	1000	10000	1000	1	1	1	1	1000	10000	10000	1000
1	0	0	0	0	0	0	1	1	1	1000	1000	1000	0	0	0	0	0	0	1000	1000	1000
1	0	0	0	0	0	0	1	1	1	1000	1000	1000	0	0	0	0	0	0	1000	1000	1000
1	0	0	0	0	0	1	1	1	1	1000	1000	1000	0	0	0	0	0	10	1000	1000	1000

This material, provided by the authors as a supplement to the article, is not part of Emerging Infectious Diseases contents.

1	1	0	0	0	1	1	1	1	1	100	100	100	10	0	0	0	100	1000	1000	100	100
0	1	1	0	0	0	0	0	0	0	0	0	0	10	10	0	0	0	0	0	0	0
0	1	1	1	1	1	1	1	0	0	0	0	0	1000	1000	100	100	10000	1000	100	0	0
0	1	1	1	1	1	1	1	0	0	0	0	0	1000	1000	1000	1000	10000	10000	1000	0	0
1	1	0	0	0	1	1	1	1	1	10	10	100	100	0	0	0	10	1000	1000	10	10
1	1	0	0	0	0	1	1	1	1	10	10	100	100	0	0	0	0	1000	1000	1000	10
1	1	1	1	1	1	1	1	1	1	100	100	10000	1000	1	1	1	1	10000	10000	100	100
1	1	1	0	1	1	1	0	0	0	0	0	10	1000	1000	0	10	100	100	0	0	0
1	1	1	1	1	1	1	1	0	0	0	0	10000	10000	1000	1	1000	10000	10000	100	0	0
0	1	1	1	1	1	1	0	0	0	0	0	0	1000	1000	100	1000	1000	1000	0	0	0
1	1	1	0	1	1	1	1	0	0	0	0	100	100	100	0	100	1000	1000	100	0	0
0	1	0	0	0	1	1	1	1	0	0	0	0	1	0	0	0	1	1	1	1	0
1	1	1	1	1	1	1	1	1	1	1000	1000	1000	1000	1000	1000	1000	1000	1000	1000	1000	1000
0	1	1	0	0	1	1	1	0	0	0	0	0	100	100	0	0	100	100	100	0	0
0	1	1	1	1	1	1	1	0	0	0	0	0	10	10	10	10	10	10	1	0	0
0	1	1	1	1	1	1	0	0	0	0	0	0	100	1000	1000	1000	1000	100	0	0	0
0	1	1	0	1	1	1	0	0	0	0	0	0	100	100	0	10	100	100	0	0	0
1	1	1	1	1	1	1	1	1	1	1000	1000	1000	1000	1000	1000	1000	1000	1000	1000	1000	1000
1	1	1	1	1	1	1	1	1	1	1	1	100	1000	1000	1000	1000	1000	1000	100	1	1
0	1	1	1	1	1	1	1	0	0	0	0	0	100	100	10	100	100	100	10	0	0
1	1	1	1	1	1	1	1	1	1	10000	10000	10000	10000	10000	10000	10000	10000	10000	10000	10000	10000
0	1	1	1	1	1	1	1	0	0	0	0	0	1000	1000	10	1000	10000	10000	1000	0	0
0	1	1	1	0	1	1	1	0	0	0	0	0	1000	1000	100	0	10000	10000	1000	0	0
0	1	1	0	0	1	1	1	0	0	0	0	0	1	1	0	0	10	10	10	0	0
1	1	1	1	1	1	1	1	1	1	1000	1000	10000	10000	10000	10000	10000	10000	10000	10000	1000	1000
1	1	1	1	1	1	1	1	1	1	1	1	100	100	100	100	100	100	100	1	1	1
1	1	1	1	1	1	1	1	1	1	1000	1000	1000	1000	1000	1000	1000	1000	1000	1000	1000	1000
1	1	1	1	1	1	1	1	1	1	1000	1000	1000	1000	1000	1000	1000	1000	1000	1000	1000	1000
0	1	1	1	1	1	1	1	0	0	0	0	0	10000	10000	100	100	10000	10000	1	0	0
0	1	1	1	1	1	1	1	0	0	0	0	0	1000	1000	100	100	1000	1000	100	0	0
1	1	1	1	1	1	1	1	1	1	1000	1000	1000	1000	1000	1000	1000	1000	1000	1000	1000	1000
1	1	1	1	1	1	1	1	1	1	1000	1000	1000	1000	1000	1000	1000	1000	1000	1000	1000	1000
1	0	0	0	0	0	1	1	1	1	100	100	100	0	0	0	0	0	1000	1000	100	100
1	1	1	1	1	1	1	1	1	1	10000	10000	10000	10000	10000	10000	10000	10000	10000	10000	10000	10000
1	1	1	1	1	1	1	1	1	1	10000	10000	10000	10000	10000	10000	10000	10000	10000	10000	10000	10000
1	0	0	0	0	0	0	0	1	1	1	1	1	0	0	0	0	0	0	0	1	1

1	1	0	0	0	0	1	1	1	1	10	10	10	10	0	0	0	0	10	10	10	10
1	1	1	1	1	1	1	1	1	1	1000	1000	1000	1000	1000	1000	1000	1000	1000	1000	1000	1000
1	1	1	1	1	1	1	1	1	1	100	1000	1000	100	1	1	1	1	100	1000	1000	100
0	1	1	1	1	1	1	0	0	0	0	0	0	1000	1000	1000	1000	1000	100	0	0	0
0	1	1	1	1	1	1	0	0	0	0	0	0	100	100	10	100	100	100	0	0	0
0	1	1	1	1	1	1	0	0	0	0	0	0	1	1	1	1	1	1	0	0	0
0	0	0	0	0	0	0	0	1	1	1	1	0	0	0	0	0	0	0	0	1	1
1	1	1	1	1	1	1	1	1	1	100	100	100	100	100	100	100	100	100	100	100	100
1	1	1	0	1	1	1	1	0	0	0	0	10	100	100	0	100	100	100	1	0	0
1	1	1	1	1	1	1	1	1	1	1000	1000	1000	1000	1000	1000	1000	1000	1000	1000	1000	1000
1	1	1	1	1	1	1	1	1	1	10	10	10	10	10	10	10	10	10	10	10	10
1	1	1	1	1	1	1	1	1	1	100	100	100	100	100	100	100	100	100	100	100	100
1	1	1	1	1	1	1	1	1	1	1000	1000	100	100	100	100	100	100	100	1000	1000	1000
0	0	0	0	0	0	0	1	1	1	10	10	0	0	0	0	0	0	0	10	10	10
1	1	1	1	1	1	1	1	1	1	10000	10000	10000	10000	10000	10000	10000	10000	10000	10000	10000	10000
1	1	1	1	1	1	1	1	1	1	10	10	10	10	10	10	10	10	10	10	10	10
1	1	1	1	1	1	1	1	1	1	10000	10000	10000	10000	10000	10000	10000	10000	10000	10000	10000	10000
0	0	0	0	0	0	0	0	1	1	1	1	0	0	0	0	0	0	0	0	1	1
0	0	0	0	0	0	0	0	0	0	0	0	0	0	0	0	0	0	0	0	0	0
1	1	1	1	1	1	1	1	1	1	10000	10000	10000	10000	10000	10000	10000	10000	10000	10000	10000	10000
1	1	1	1	1	1	1	1	1	1	10000	10000	10000	10000	10000	10000	10000	10000	10000	10000	10000	10000
1	1	1	1	1	1	1	1	1	1	1000	1000	1000	1000	1000	1000	1000	1000	1000	1000	1000	1000
1	0	0	0	0	0	0	1	1	1	1	1	1	0	0	0	0	0	0	1	1	1
1	1	0	0	0	0	1	1	1	1	1000	1000	1000	100	0	0	0	0	100	1000	1000	1000
1	0	0	0	0	0	0	1	1	1	10	10	10	0	0	0	0	0	0	10	10	10
1	1	1	1	1	1	1	1	1	1	100	100	1000	100	100	100	100	100	100	1000	100	100
1	0	0	0	0	0	0	1	1	1	1000	1000	100	0	0	0	0	0	0	100	1000	1000
1	1	1	1	1	1	1	1	1	1	10000	10000	10000	1000	100	100	100	100	100	10000	10000	10000
1	1	1	1	1	1	1	1	1	1	1000	1000	1000	100	100	100	100	100	100	1000	1000	1000
1	1	0	0	0	0	0	1	1	1	100	100	100	100	0	0	0	0	0	100	100	100
1	1	1	1	1	1	1	1	1	1	10000	10000	10000	1000	1000	1000	1000	1000	1000	10000	10000	10000
0	0	0	0	0	0	0	1	1	1	10	0	0	0	0	0	0	0	0	10	10	10
0	0	0	0	1	1	1	1	1	1	10	10	0	0	0	0	10	10	10	10	10	10
1	1	1	1	1	1	1	1	1	1	1000	1000	1000	1000	1000	1000	1000	1000	1000	1000	1000	1000
1	1	1	1	1	1	1	1	1	1	10000	10000	10000	1000	1000	1000	1000	1000	1000	10000	10000	10000
0	1	1	1	1	1	1	0	0	0	0	0	0	1000	1000	1	1	100	1000	0	0	0

This material, provided by the authors as a supplement to the article, is not part of Emerging Infectious Diseases contents.

1	0	0	0	0	0	0	0	1	1	1000	1000	1000	0	0	0	0	0	0	0	1000	1000
1	1	1	1	1	1	1	1	1	1	10	10	10	10	10	10	10	10	10	10	10	10
1	1	1	1	1	1	1	1	1	1	10000	10000	1000	1000	1000	1000	1000	1000	1000	1000	10000	10000
0	0	0	0	0	0	0	0	1	1	1	1	0	0	0	0	0	0	0	0	1	1
0	0	0	0	0	0	0	0	1	1	1	1	0	0	0	0	0	0	0	0	1	1
